# Extremely elevated serum alkaline phosphatase level upon treatment with teriparatide: a case report

**DOI:** 10.1186/s13256-020-02416-7

**Published:** 2020-07-03

**Authors:** Ali Javinani, Hamid Reza Aghaei Meybodi, Hoda Kavosi

**Affiliations:** 1 Rheumatology Research Center, Tehran University of Medical Sciences (TUMS), PO-Box: 1411713137, Tehran, Iran; 2 Endocrinology and Metabolism Research Center, Endocrinology and Metabolism Research Institute, Tehran University of Medical Sciences, Tehran, Iran

**Keywords:** Glucocorticoid-induced osteoporosis, Systemic lupus erythematosus, Teriparatide

## Abstract

**Background:**

Teriparatide is a homolog of human parathyroid hormone (1–34), which is approved for the treatment of postmenopausal and glucocorticoid-induced osteoporosis. Several minor and transient side effects have been reported for teriparatide. However, controversial findings showed an increased risk of more significant adverse effects, including osteosarcoma in humans, although this finding has been demonstrated primarily in murine models.

**Case presentation:**

We present a case of a 22-year-old Persian man with a previous history of systemic lupus erythematosus and glucocorticoid-induced osteoporosis. He had a previous history of joint hypermobility, idiopathic kyphoscoliosis, mitral valve prolapse, and bilateral congenital inguinal hernia, which were probably compatible with an inherited connective tissue disease. He was treated with teriparatide for 7 months because of glucocorticoid-induced osteoporosis. He was referred with a complaint of generalized bone pain and an extremely elevated serum alkaline phosphatase concentration of 6480 U/L (normal range, 80–306). A whole-body bone scan revealed a diffuse increased osseous uptake. Furthermore, the patient’s systemic lupus erythematosus was clinically inactive on the basis of laboratory findings during this period. The medication was discontinued, and the patient’s serum alkaline phosphatase level began to decline.

**Conclusions:**

To the best of our knowledge, this is the first case of an osteoblast hyperactivation state observed during treatment with teriparatide. It appears that the osteoblastogenic effect of teriparatide might induce this condition and, most likely, osteosarcoma in certain populations. However, the potential influence of the patient’s young age, systemic lupus erythematosus, underlying inherited connective tissue disease, and medication use cannot be ignored. The potential risk factors of this side effect shall be studied in specific subpopulations of patients with osteoporosis in future studies.

## Background

Teriparatide is the biosynthetic human parathyroid hormone (PTH) (1–34) that is classified as the anabolic agent used for osteoporosis treatment [1]. In contrast to antiresorptive drugs, teriparatide induces bone formation by activating osteoblasts and reducing their apoptosis. Teriparatide is approved for the treatment of postmenopausal and glucocorticoid-induced osteoporosis (GIOP) at a daily dosage of 20 μg via subcutaneous injection. Several minor and negligible side effects of teriparatide have been reported, including injection site reactions and transient hypercalcemia, which is generally tolerated by patients. However, anecdotal results present more significant side effects, including an elevated risk of osteosarcoma [2]. Such findings are strongly supported by studies in the murine model following the 2-year treatment of rats with teriparatide [3]. Accordingly, teriparatide should be avoided in high-risk patients, including individuals with a history of previous radiation, malignancy, bone metastasis, and Paget disease of the bone. In this report, we present a case of a patient with systemic lupus erythematosus (SLE) and GIOP with an acutely elevated level of serum alkaline phosphatase after 7 months of treatment with teriparatide.

## Case presentation

A 22-year-old Persian man was referred to our outpatient SLE clinic for regular follow-up on 27 December 2018. He complained of severe and progressive generalized bone pain that he had begun experiencing 2 weeks before his admission. He did not report any clinical symptoms in favor of SLE flare-up. His physical examination revealed generalized bone tenderness over the sternum, vertebra, and pelvis. His joints were normal without any sign of arthritis. He weighed 69 kg and was 170 cm tall (body mass index, 23.87 kg/m^2^). The results of the rest of his examinations were unremarkable. The patient’s vital signs were within the normal range, and the results of examinations of his mucocutaneous, cardiopulmonary, and neurologic systems were normal.

However, the laboratory examinations showed an exceedingly elevated level of serum alkaline phosphatase (ALP) of 3609 U/L (reference range, 40–130). The rest of the tests consisted of a complete blood count, erythrocyte sedimentation rate, C-reactive protein, liver function, glucose and lipid profiles, serum creatinine, urinalysis, and anti-double-stranded deoxyribonucleic acid (DNA); all of these were within the normal ranges. The last laboratory examination was performed on 15 November 2018 and indicated a normal serum ALP level of 141 U/L.

The patient’s medical history was significant for an antecedent of SLE from 2 years earlier, which was diagnosed with the primary manifestations of oral ulcers, polyarthritis, hematuria, and proteinuria, along with positive antinuclear antibody and diminished complement level. A renal biopsy was performed at the time of diagnosis; this biopsy was compatible with mesangial proliferative lupus nephritis (class II). At this time, hydroxychloroquine (400 mg/day), prednisolone (15 mg/day), azathioprine (2.5 mg/kg/day), and calcium vitamin D supplementation began being administered. According to the patient’s gastrointestinal side effects and persistent dysmorphic hematuria, azathioprine was replaced with mycophenolate mofetil (2 g/day), and prednisolone was tapered to 5 mg/day from 1 year prior.

The patient’s bone mineral densitometry (BMD) was assessed at the time of the diagnosis and again one year later using the same instrument (Hologic, Marlborough, MA, USA) (Table 1). At the time of diagnosis, the patient had osteopenia (lumbar spine Z-score, − 2.2), which was unjustifiable on the basis of his age. The results of laboratory examinations, including serum electrolytes and endocrine panel evaluations, were normal (Table 1). However, the patient’s serum ALP level was lower than normal at 175 U/L (normal range, 245–768). Supplemental calcium vitamin D was administered, and the patient was referred to a sports medicine specialist.

**Table 1:** Laboratory studies

March 2017			December 2018		
Lab test	Value	Normal range	Lab test	Value	Normal range
FSH	1.3 mU/ml	0.95–11.9	AST	12 U/L	Up to 37
LH	3.0 mU/ml	0.57–12.0	ALT	13 U/L	Up to 41
Testosterone	4.7 ng/ml	2.4–8.7	**ALP**	**6480 U/L**	80–306
Cortisol at 0800 hours	9.7 μg/ml	3.7–19.4	GGT	8 U/L	8–61
TSH	1.65 mU/L	0.27–4.2	LDH	231 U/L	< 250
iPTH	26.96 pg/ml	15–65	iPTH	24.1 pg/ml	15–65
Calcium	9.6 mg/dl	8.6–10.2	Calcium	9.4 mg/dl	8.6–10.2
Phosphate	4.0 mg/dl	2.5–4.5	Phosphate	3.4 mg/dl	2.5–4.5
25(OH)-vitamin D	53.9 ng/ml	> 20	25 (OH) vitamin D	34.7 ng/ml	> 20
Creatinine	0.9 mg/dl	0.7–1.2	Uric acid	5.2 mg/dl	3.4–7.0
SPEP	Normal		Urine Ca/Cr	0.044	< 0.21

Values outside of the normal range are shown in bold type

*Abbreviations: ALT* Alanine aminotransferase, *AST* Aspartate aminotransferase, *FSH* Follicle-stimulating hormone, *GGT* γ-Glutamyltransferase, *iPTH* Parathyroid hormone, *LDH* Lactate dehydrogenase, *LH* Luteinizing hormone, *SPEP* Serum protein electrophoresis, *TSH* Thyroid-stimulating hormone

A detailed investigation of the patient’s past medical history revealed instances of idiopathic kyphoscoliosis, mitral valve prolapse, and a bilateral congenital inguinal hernia that was operated on in the infantile period. The patient’s physical examination was compatible with hypermobility of the joints (Beighton score, 4). There was no evidence to suggest delayed or absent puberty. The results of the rest of the investigations, including a complete endocrine panel and ocular evaluation, were normal. It was speculated that the patient had an occult connective tissue disease such as Ehlers-Danlos syndrome. However, due to the unavailability of genetic studies and the lack of full-blown manifestations of inherited collagen disease, this diagnosis remained unidentified.

According to the very low bone mass of the patient and diminished bone density at the hip and the lumbar spine (Table 1), subcutaneous injection of teriparatide (Forteo® 20 μg/day; Eli Lilly, Indianapolis, IN, USA) was started on 2 May 2018. Additionally, he was receiving hydroxychloroquine 200 mg/day, prednisolone 5 mg/day, mycophenolate mofetil 500 mg/day, and calcium vitamin D supplementation. He denied any use of other medications, even over-the-counter medicines and herbal remedies. He tolerated the teriparatide well without any adverse drug reactions and was strictly adherent to his medications and physical activities. His family history was remarkable for SLE in his mother. His history was negative for cigarette smoking and any drug abuse.

**Fig. 1:**
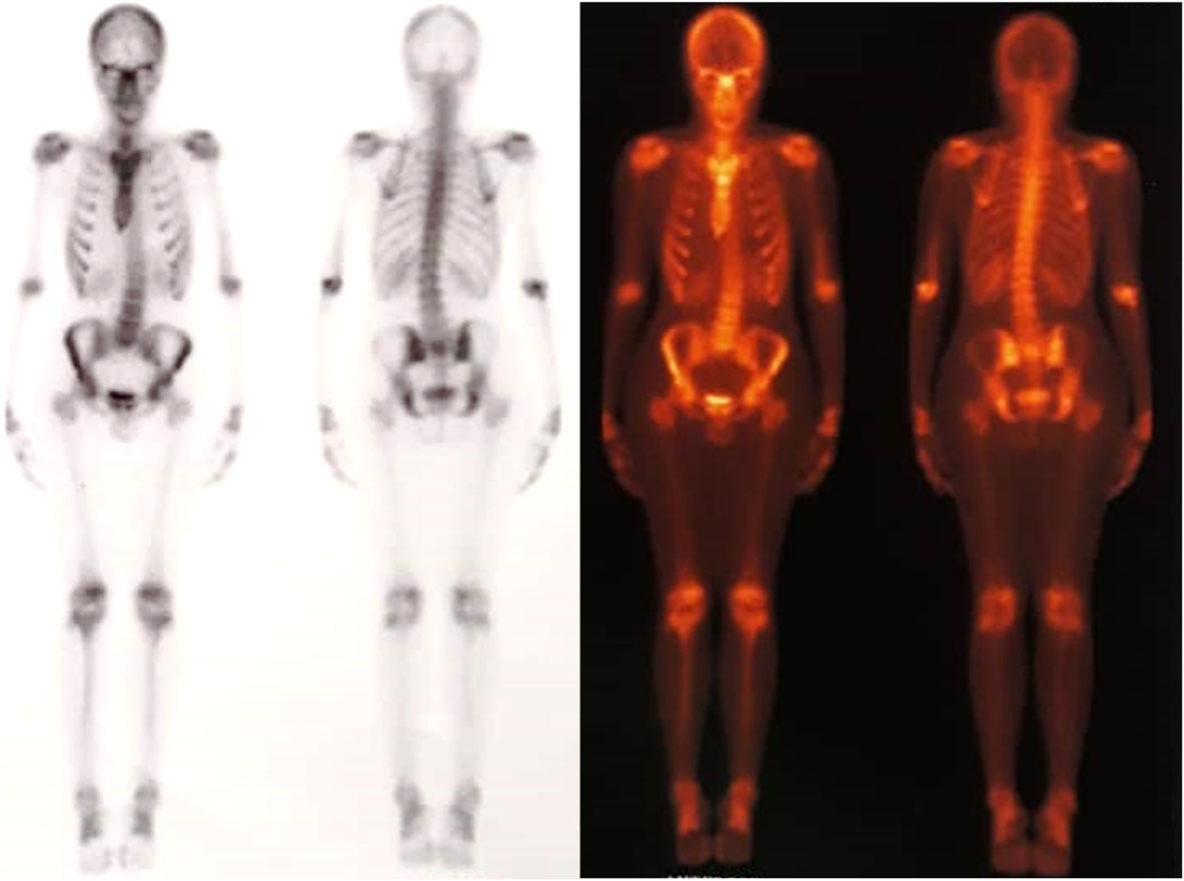
Whole-body bone scan demonstrating metabolic bone disease

**Fig. 2:**
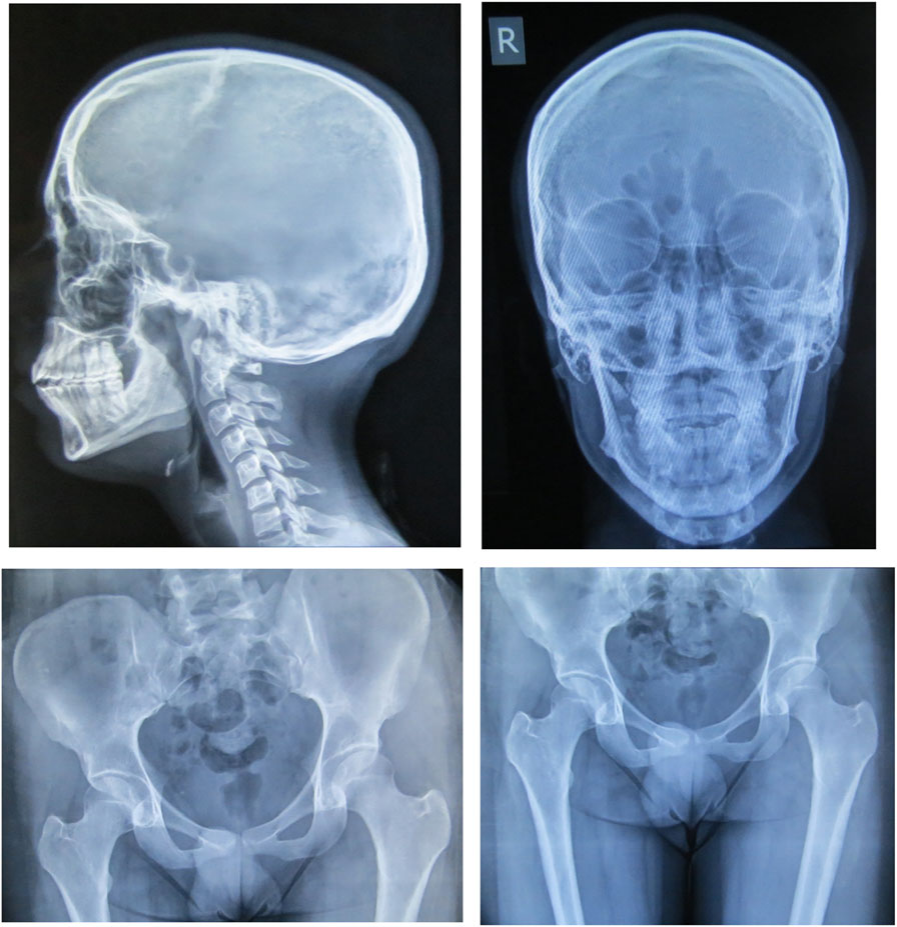
Bone survey of skull, hip, and femur. X-ray finding was normal

The workup began for this patient according to his generalized bone pain and tenderness and extremely elevated level of serum ALP. The results of laboratory examinations done on 31 December 2018 are shown in Table 1. The serum and urinary bone turnover marker measurement kits were unavailable at that moment, and the patient did not consent to undergo bone biopsy. His whole-body bone scan showed a superscan pattern with diffusely increased osseous uptake in the calvarium, supraorbital crests, and mandible (Lincoln sign), as well as all the costochondral joints, both sacroiliac joints (butterfly sign), and the pubic symphysis (Fig. 1). Most of the epiphyseal plates also showed a significant diffuse and symmetric uptake. The pattern of the scan was compatible with metabolic bone disease (MBD) associated with a diffuse bone formation state without any evidence for fracture, bone metastasis, or Paget disease. Abdominopelvic ultrasonography and skull and pelvic x-ray results were entirely normal (Fig. 2).

Briefly, we had a patient complaining of recent-onset generalized bone pain and tenderness in addition to a newly raised serum ALP level. The patient’s γ-glutamyltransferase level, liver function test, and biliary system ultrasonography were normal. His whole-body bone scan was in favor of MBD without any evidence of bone tumors. Neither the patient’s clinical manifestations nor his laboratory test results were compatible with SLE flare-up. Taken together, all of these manifestations were probably related to teriparatide use, and the medication was discontinued on 31 December 2018. The patient’s serum ALP level began to decline with the level of 6423 U/L (normal range, 80–306) on 10 January 2019, to 3492 U/L (80–306) on 18 January 2019, 598 U/L (40–130) on 24 January 2019, 151 U/L (40–130) on 10 February 2019, 40 U/L (40–130) on 18 June 2019, and 42 U/L (40–130) on 2 March 2020. The calcium supplement dosage was augmented to prevent probable hungry bone syndrome. However, the serum calcium and phosphorus levels were strictly normal during this period. Most of the laboratory examinations were performed in a single laboratory, whereas the abnormal findings were also rechecked in another medical center. This could justify the enormously different ALP levels in a short time interval. The clinical manifestations completely resolved, and the patient did not experience any SLE relapse. The third BMD was performed on 8 April 2019 with a similar machine, revealing a significant increase of bone density (Table 2). After 7 months of treatment with teriparatide, the bone density of the lumbar spine, femoral neck, total hip, and one-third distal radius was increased 4.5%, 4.8%, 23%, and 6.1%, respectively. To the best of our knowledge, this is the highest rate of increased bone density in such a short period of time reported in the literature.

**Table 2:** Bone mineral densitometry of the patient

Region	23 March 2017 SLE diagnosis		15 March 2018 Before teriparatide commencement		8 April 2019 4 months after discontinuing teriparatide	
	Z-score	BMD (g/cm^2^)	Z-score	BMD (g/cm^2^)	Z-score	BMD (g/cm^2^)
Lumbar spine	− 2.2	0.852	− 2.8	0.781	− 2.5	0.816
Femoral neck	− 0.8	0.827	− 1.1	0.786	− 0.8	0.824
Total hip	− 0.9	0.894	− 1.8	0.759	− 0.6	0.934
One-third distal radius	–	–	− 3.2	0.648	− 2.4	0.688

*BMD* Bone mineral densitometry, *SLE* Systemic lupus erythematosus

## Discussion and conclusion

MBD is a general term that refers to the group of disorders that affect bone remodeling, which is usually reversible after eliminating the causative agent. Most of these disorders are characterized by an abnormal rate of bone formation, resorption, or mineralization. In our patient, the increased uptake of radiotracers in the whole-body bone scan, elevated ALP, and increased bone density were probably indicative of an osteoblast hyperactivation state that could be a member of MBD. In addition, the discontinuation of teriparatide led to a rapid decline in serum ALP levels, which further increased the likelihood that this state was entirely related to teriparatide. Moreover, the patient’s bone density was significantly increased after teriparatide stoppage, thus showing its obvious influence on his osteoporosis treatment. The SLE was clinically inactive on the basis of laboratory examinations. The patient’s renal, hepatobiliary, and endocrine profiles were normal, and all interactions between teriparatide and medications that were used by the patient have been reported. Consequently, it appeared that the osteoblast hyperactivation state in our patient was solely owing to teriparatide use. However, the probable role of quiescent SLE (an occult inherited connective tissue disorder), his medications, and his young age in the development of this side effect could not be proved or rejected.

Similar to our patient’s case, Hajime *et al.* reported a case of a 49-year-old woman with intermittent porphyria and GIOP [4]. The teriparatide was started with a dosage of 20 μg/day according to her unexplained tibia fracture. Two weeks after treatment commencement, she developed hypercalcemia, hypophosphatemia, and elevated ALP level (634 U/L). These values became normal after 10 weeks of teriparatide cessation. The authors speculated that this adverse effect was related to the underlying intermittent porphyria disorder. Taken together, it appears that future studies of teriparatide in a specific population of patients, including those with genetic and autoimmune disorders, could help to elucidate this issue.

The probable osteoblast hyperactivation state in our patient is related to the potent effect of teriparatide on osteoblasts that mediates its anabolic role in osteoporosis treatment. The PTH could induce osteoblast formation, development, and activation by several different pathways [5]. PTH can directly induce a mitogenic effect on osteoblasts and inhibit their apoptosis via the PTH-1 receptor. In addition, PTH can induce the bone ALP via the β-catenin activation that enhances osteoblast differentiation [6]. Likewise, it is also demonstrated that teriparatide treatment increases the level of bone ALP and promotes the maturation of osteoblast precursors [7]. Taken together, the vigorous influence of teriparatide on the expression of bone ALP and osteoblast maturation could probably justify the development of this hyperactivation state in our patient.

Preliminary murine studies revealed the elevated incidence of osteosarcoma in Sprague-Dawley rats treated with a teriparatide dose of 13.6 μg/kg/day for 2 years [3]. Watanabe *et al.* reported the noncarcinogenic dose of 4.5 μg/kg/day for male and female rats, which is far beyond the therapeutic dose used in humans (20 μg/day) [3]. However, a small number of osteosarcoma cases have been reported in humans treated with teriparatide [2]. Nonetheless, an extremely low number of cases among so many individuals taking teriparatide makes the osteosarcoma frequency similar in the general population. In addition, the Forteo Patient Registry, which consists of 242,782 person-years of follow-up, did not demonstrate any case with osteosarcoma [8]. Although the association of teriparatide and osteosarcoma has not been established yet, precautions should be considered for high-risk individuals. The U.S. Food and Drug Administration has warned that teriparatide is not suitable for children with open epiphyses [9]. According to the results of the whole-body bone scan of our patient, the patient’s epiphyseal plates had taken up radiotracers that might cause the incomplete closure of his epiphyses. This finding significantly necessitates serious precautions in administrating teriparatide in children and young adults.

In conclusion, we report an extremely elevated serum ALP level after 7 months of treatment with teriparatide in a young adult patient with GIOP, SLE, and probable underlying connective tissue disease. The osteoblast hyperactivation state was diagnosed on the basis of generalized bone pain, diffuse increased osseous uptake on the bone scan, and extremely elevated serum ALP levels. The clinical and laboratory findings returned to normal 2 months after teriparatide use had been discontinued. However, the potential influence of the patient’s young age, SLE, underlying connective tissue disease, and concomitant medications on teriparatide’s side effects should be investigated in future studies.

## Data Availability

Not applicable.

## References

[1] Compston JE, McClung MR, Leslie WD. Osteoporosis. Lancet. 2019;393(10169):364–76. 10.1016/S0140-6736(18)32112-3 30696576

[2] Subbiah V, Madsen VS, Raymond AK, Benjamin RS, Ludwig JA. Of mice and men: divergent risks of teriparatide-induced osteosarcoma. Osteoporosis Int. 2010;21(6):1041–5. 10.1007/s00198-009-1004-0 19597911

[3] Watanabe A, Yoneyama S, Nakajima M, Sato N, Takao-Kawabata R, Isogai Y, *et al.* Osteosarcoma in Sprague-Dawley rats after long-term treatment with teriparatide (human parathyroid hormone (1-34)). J Toxicol Sci. 2012;37(3):617–29. 10.2131/jts.37.617 22688001

[4] Hajime M, Okada Y, Mori H, Tanaka Y. A case of teriparatide-induced severe hypophosphatemia and hypercalcemia. J Bone Miner Metab. 2014;32(5):601–4. 10.1007/s00774-014-0564-z 24553859

[5] Canalis E. Management of endocrine disease: novel anabolic treatments for osteoporosis. Eur J Endocrinol. 2018;178(2):R33–44. 10.1530/EJE-17-0920 PMC5819362 29113980

[6] Tian Y, Xu Y, Fu Q, He M. Parathyroid hormone regulates osteoblast differentiation in a Wnt/β-catenin-dependent manner. Mol Cell Biochem. 2011;355(1–2):211–6. 10.1007/s11010-011-0856-8 21533763

[7] D’Amelio P, Tamone C, Sassi F, D’Amico L, Roato I, Patane S, *et al.* Teriparatide increases the maturation of circulating osteoblast precursors. Osteoporosis Int. 2012;23(4):1245–53. 10.1007/s00198-011-1666-2 21617993

[8] Gilsenan A, Harding A, Kellier-Steele N, Harris D, Midkiff K, Andrews E. The Forteo Patient Registry linkage to multiple state cancer registries: study design and results from the first 8 years. Osteoporosis Int. 2018;29(10):2335–43. 10.1007/s00198-018-4604-8 PMC6154045 29978254

[9] Food and Drug Administration’s Office of Pediatric Therapeutics and Pediatric and Maternal Health Staff. Forteo not indicated in children with open epiphyses. AAP News. 2010;31(1):12.

